# Methods for the Clinical Validation of Digital Endpoints: Protocol for a Scoping Review Abstract

**DOI:** 10.2196/47119

**Published:** 2023-10-26

**Authors:** Sílvia Rego, Ana Rita Henriques, Sofia Silvério Serra, Teresa Costa, Ana Maria Rodrigues, Francisco Nunes

**Affiliations:** 1 Fraunhofer Portugal Research Center for Assistive Information and Communication Solutions Porto Portugal; 2 Faculty of Medicine University of Porto Porto Portugal; 3 NOVA Medical School Universidade NOVA de Lisboa Lisbon Portugal

**Keywords:** digital endpoint, digital biomarker, mobile health technologies, mobile health, mHealth, remote monitoring, wearable technology, scoping review, review method, validate, validation, outcome measure, sensor, wearable

## Abstract

**Background:**

Clinical trials often use digital technologies to collect data continuously outside the clinic and use the derived digital endpoints as trial endpoints. Digital endpoints are also being developed to support diagnosis, monitoring, or therapeutic interventions in clinical care. However, clinical validation stands as a significant challenge, as there are no specific guidelines orienting the validation of digital endpoints.

**Objective:**

This paper presents the protocol for a scoping review that aims to map the existing methods for the clinical validation of digital endpoints.

**Methods:**

The scoping review will comprise searches from the electronic literature databases MEDLINE (PubMed), Scopus (including conference proceedings), Embase, IEEE (Institute of Electrical and Electronics Engineers) Xplore, ACM (Association for Computing Machinery) Digital Library, CENTRAL (Cochrane Central Register of Controlled Trials), Web of Science Core Collection (including conference proceedings), and Joanna Briggs Institute Database of Systematic Reviews and Implementation Reports. We will also include various sources of gray literature with search terms related to digital endpoints. The methodology will adhere to the Joanna Briggs Institute Scoping Review and the Guidance for Conducting Systematic Scoping Reviews.

**Results:**

A search for reviews on the existing evidence related to this topic was conducted and has shown that no such review was previously undertaken. This review will provide a systematic assessment of the literature on methods for the clinical validation of digital endpoints and highlight any potential need for harmonization or reporting of methods. The results will include the methods for the clinical validation of digital endpoints according to device, digital endpoint, and clinical application goal of digital endpoints. The study started in January 2023 and is expected to end by December 2023, with results to be published in a peer-reviewed journal.

**Conclusions:**

A scoping review of methodologies that validate digital endpoints is necessary. This review will be unique in its breadth since it will comprise digital endpoints collected from several devices and not focus on a specific disease area. The results of our work should help guide researchers in choosing validation methods, identify potential gaps in the literature, or inform the development of novel methods to optimize the clinical validation of digital endpoints. Resolving these gaps is the key to presenting evidence in a consistent way to regulators and other parties and obtaining regulatory acceptance of digital endpoints for patient benefit.

**International Registered Report Identifier (IRRID):**

PRR1-10.2196/47119

## Introduction

### Background

Digital technologies such as smartphones, wearables, implantables, digestibles, and other biosensors present new opportunities to collect and analyze health data [1,2]. They enable more accurate and reliable identification and quantification of different aspects of one’s health [3]. A wide range of parameters can be collected by digital devices such as physiological, anatomic, and pathological, as well as behavioral-, social-, or activity-related characteristics [3]. The patient can use the device in everyday life while data are passively collected or can perform assessments at the clinic [4]. Before clinicians and researchers can use data acquired by digital technologies, algorithms transform the data into metrics designated as digital endpoints [4].

In clinical care, digital endpoints may increase diagnostic accuracy and improve treatment decisions, as the clinicians can be provided with more information about the patient’s status and treatment response in everyday life [2]. In clinical trials, digital endpoints can be used as replacements or as a proxy for outcome measures. This new type of endpoint consists not only of traditional endpoints assessed in a new way but also endpoints that were not previously possible to obtain [5]. By being collected automatically outside the clinic, digital endpoints enable more frequent or continuous data collection while requiring fewer staff and fewer clinic visits and reducing the need for patients to respond to so many questionnaires [6-9]. Despite the concerns that digital literacy may restrict patients’ access to clinical trials [8], many argue that technology can support increased trial participation and retention [6]. In addition, it is not consensual whether using digital endpoints will increase the efficiency and effectiveness of clinical trials [1]. Regardless of these concerns, pharmaceutical and technological companies have turned their attention toward digital endpoints, incorporating them into clinical trials as study endpoints [10,11]. So far, 436 clinical trials using digital endpoints have been registered in the Digital Medicine Society Library of Digital Endpoints, out of which 205 (47%) consisted of drug studies, 175 (40.1%) were device-related studies, 30 (6.9%) were biological products, 1 (0.2%) was genetic, and 25 (5.7%) were other medical products [3].

Digital endpoints have yet to be accepted to support new medical product (or new applications of medical products) approvals [12]. There appears to be a global regulatory consensus on using digital devices in clinical trials [1,7,12], but only validated digital endpoints will be suitable for supporting safety and efficacy claims in applications to regulatory authorities [13]. The same applies to digital endpoints intended to be medical products [13]. However, the current need for a well-defined methodology for the clinical validation of digital endpoints poses a critical limitation [13].

The V3 framework, which combines software and clinical development, establishes the foundation for evaluating digital clinical endpoints [13]. Clinical validation is defined in the V3 framework as an evaluation of whether digital endpoints “acceptably identifies, measures or predicts a meaningful clinical, biological, physical, functional state, or experience, in the stated context of use (which includes a specified population)” and takes place after both the verification and analytical validation processes [13]. This assessment evaluates the association between a digital endpoint and a clinical condition. It is subject to similar principles of research design and statistical analysis of clinical validation of traditional tests, tools, and measurement instruments [13,14]. In general terms, clinical validation comprises the assessment of content validity, reliability, and accuracy (which validates the digital endpoint against a gold standard) and the establishment of meaningful thresholds [12,15].

Some approaches have been suggested, such as the Food and Drug Administration Guidance on patient-reported outcomes [16] or the Clinical Trials Transformation Initiative Recommendations for endpoints generated by mobile technology [5], but no standardized framework for clinical validation is currently available. Standardization is challenging; indeed, there is a wide variety of digital endpoints [13]. If we consider bodily functions captured, a few examples include diverse parameters such as heart rate, cognition, lung function, and gait. If we divide digital endpoints by type of devices, we have, for example, smartphones, wearables, implantables, and digestibles. Adding to that, and similarly to traditional endpoints, the clinical goal of the digital endpoints (diagnostic, safety, response, monitoring, prognostic, risk, and predictive) also determines variations in methods of clinical validation.

A search for reviews on the existing evidence related to this topic was conducted and has shown that no such review was previously undertaken. This study aims to map the literature on the methods for validating digital endpoints used or proposed. A scoping review is a suitable approach to synthesize such a complex topic comprehensively [17-19]. We hypothesize that in a scoping review, important patterns of methods for clinical validation of digital endpoints grouped by bodily functions, type of devices, and clinical application goal will emerge. The results of this work will guide researchers and identify potential gaps in the literature, which in turn can inform the development of novel methods to optimize the clinical validation of digital endpoints.

### Review Question

The main research question addressed by the scoping review will be “What methodologies have been employed or proposed for clinical validation of digital endpoints?”

## Methods

### Overview

This protocol is reported in line with the Joanna Briggs Institute (JBI) Scoping Review Protocol [8]. The scoping review will be conducted and reported following the methodology for scoping reviews by the JBI Scoping Review [8] and the Guidance for Conducting Systematic Scoping Reviews [9].

### Search Strategy

An exploratory search of MEDLINE (PubMed) was performed to retrieve sentinel papers and identify text words contained in the title and abstract and the index terms in the description of those papers to compile a list of terms to inform our search strategy. The search strategy for PubMed was drafted by the first author and further refined through team discussion with the remaining authors. In total, 2 librarians translated and adapted the search strategy for other electronic databases and ran the searches. The final search strategy is reported in Multimedia Appendix 1.

We ran the search query in MEDLINE (PubMed), Scopus (including conference proceedings), Embase, IEEE (Institute of Electrical and Electronics Engineers) Xplore, ACM (Association for Computing Machinery) Digital Library, CENTRAL (Cochrane Central Register of Controlled Trials), Web of Science Core Collection (including conference proceedings), and JBI Database of Systematic Reviews and Implementation Reports. We included databases indexing research related to life sciences and biomedicine and other databases of traditional engineering journals because digital medicine is a highly interdisciplinary field.

The search strategy was first developed for PubMed and then adapted to each additional database, including Boolean operators, various combinations of text words (including truncation), index terms related to digital endpoints for use in clinical practice and clinical trials, and validation studies methodologies. The search in electronic scientific databases will be supplemented by searching gray literature in Google Scholar, ClinicalTrials.gov, and at least 1 national and 2 international nonindexed conference proceedings using free text in the title and abstract related to digital endpoints and validation. This search aims to find conference papers, theses, dissertations, ongoing studies, white papers, academic and industrial reports, expert group documents, regulatory entity documents, blog posts, etc, of potential interest. These sources are essential to consider that information may be in various formats due to the novelty of the theme.

No time or language restrictions will exist, provided an English or Portuguese title and abstract are available. Relevant papers identified in languages other than English or Portuguese will be translated. Additionally, the reference lists of retrieved studies meeting the inclusion criteria will be manually searched to identify additional relevant studies. The final search results will be imported into the professional version of Rayyan [20], a web-based platform for literature review management hosted at NOVA Medical School–Universidade NOVA de Lisboa, and duplicates will be removed. In addition, we will cross-check authors’ names across gray literature and results from electronic databases to identify and remove potential duplicates.

### Study Selection and Eligibility Criteria

Study screening and selection will be performed using Rayyan [20] as it facilitates collaborative screening of papers while ensuring reviewers do not see each other’s selections. The software logs reasons for exclusion and lists disagreements between reviewers. In total, 2 reviewers will independently perform the study selection process. Blinding will be switched off for reviewers to see papers with disagreements, which will be resolved by consensus. If consensus is not possible, a third reviewer will assess the discussed abstracts or full texts and provide a decision. Reviewer agreement will be calculated using the κ coefficient and reported in the scoping review paper.

Study selection will be undertaken in 3 phases: pilot testing, title and abstract screening, and full-text review. The first phase, a pilot, will involve the screening of the same random 20 publications (title and abstracts) by all reviewers, followed by a discussion to ensure consistency and that all relevant data were captured. Any resulting changes to the inclusion and exclusion criteria will be documented and reported. In the second phase, both titles and abstracts will be assessed.

For studies to be included, they must meet the inclusion criteria (Textbox 1), which were defined based on the “Population–Concept–Context (PCC)” framework recommended by the JBI for scoping reviews [9]. Studies will be excluded if they have any characteristics listed as exclusion criteria (Textbox 1).

**Inclusion and exclusion criteria.**

**Inclusion criteria**

- Population: Human adults.
- Concept: Scientific methodologies used or proposed to validate digital clinical endpoints.
- Context: The review will include all study designs published in journals, conferences, theses, or dissertations, as well as clinical trials registered in platforms, white papers, academic and industry reports, opinion papers, blog or website or forum posts, letters, guidelines, book chapters, editorials, commentaries, papers or guidelines by an expert group, regulatory entities, or others; there will be no date and language restrictions.

**Exclusion criteria**

- Studies describing verification or analytical validation of digital endpoints.
- Studies describing clinical validation of surrogate endpoints.

In the third stage, the full texts of the selected publications will be reviewed for further assessment against the inclusion and exclusion criteria. Reasons for excluding sources of evidence at the full-text review will be recorded and reported. As an iterative approach to study selection is recommended [21], changes to this protocol may be made and reported accordingly in the scoping review.

### Data Extraction

Reviewers will independently collect data from the full text of each included study using a structured extraction form adapted by authors from the review of Polhemus et al [22] with an explanation for each data item. Our team will internally assess the appropriateness and comprehensiveness of the data extraction form. Then, following the “Updated methodological guidance for the conduct of scoping reviews” [21], all reviewers will pilot the extraction form on at least 3 (same) random studies to ensure consistency in data extraction. If a level of agreement indicated by a κ of at least 0.8 is achieved, the data extraction begins; and if not, a second (or more) round of the pilot is conducted. Modifications to the form identified through the pilot, including rewriting sentences for clarity and adding or removing items to the initial list of study characteristics, will be discussed by reviewers. The draft extraction form will include information about each included study, such as authors, year and type of source, and the key findings relevant to the review questions for original papers (Table 1) and regulatory guidelines, viewpoints, expert group recommendations, or other sources (Table 2).

**Table 1:** Preliminary data items to extract from original papers.

Data items		Explanation
**Publication details**		
	Authors and reference	Who conducted the research? Include the reference.
	Year	When was the study published?
	Type of source	In what type of literature was the study published (journal, conference, or gray literature—conference, thesis or dissertation, clinical trials registry platform, white paper, book chapter, or others)?
	Country or region	In which country or region did the study take place?
**General details**		
	Study design	What was the study’s design?
	Study aims	What were the study’s aims?
	Population	What population was studied? What were the inclusion or exclusion criteria (eg, age, disease, and disease severity)?
	Study size	How many people participated in the study?
	Sampling method	What method was used for sampling?
	Study limitations	What were the limitations of the study?
	Validation method limitations	What were the limitations of the method used for clinical validation?
**Digital endpoint details**		
	Digital endpoints	Which digital endpoints were measured? How and in what setting were the digital endpoints calculated?
	Analytical methods	How did the authors measure the relationship between clinically relevant outcomes and digital endpoints? What association measure was used?
	Clinically relevant methods	What clinically relevant endpoints were studied?
	Type of clinical application	Which type of clinical application of the digital endpoints concerning the studied outcome? Diagnostic, safety, response, monitoring, prognostic, risk, or predictive?
	Study endpoints	Was the digital endpoint used as a primary, secondary, or exploratory endpoint? What other primary, secondary, and exploratory endpoints were measured?
	The device used for the collection of digital endpoints	Which devices were used to collect digital endpoints?

**Table 2:** Preliminary data items to extract from regulatory guidelines, viewpoints, expert group recommendations, and other similar sources.

Data items			Explanation
**Publication details**			
	Authors and reference	Who wrote the paper? Include the reference.	
	Year	When was the paper published?	
	Type of source	In what type of literature was the paper published (journal, conference, gray literature—conference, thesis or dissertation, clinical trials registry platform, white paper, academic and industry report, opinion paper, blog or website or forum post, letter, guideline, book chapter, editorial, commentary, paper or guideline by expert group, regulatory entity, or others)?	
	Country or region	In which country or region was the paper written? To which regions do the recommendations apply (which country or region, global)?	
**General details**			
	Sampling size	What is the recommended or used sample size?	
	Sampling method	What method is recommended or used for sampling?	
	Study design	What study design is recommended or used for clinical validation?	
**Digital endpoint details**			
	Clinical application of digital endpoints	What is the clinical application of digital endpoints (diagnostic, safety, response, monitoring, prognostic, risk, or predictive)?	
	Type of validation methods	Which type of validation method is recommended? The trial, simulation, expert opinion, model-centered, and other?	
	Analytical methods	What methods are recommended to measure the relationship between clinically relevant outcomes and digital endpoints? What association measures should be used?	
	Analytical methods	Is there any consideration for using the digital endpoint as a primary, secondary, or exploratory endpoint?	

Further refinement may be made to the extraction form at the review stage, and the final version will be included in the scoping review. If relevant missing or additional data are necessary, the studies’ corresponding authors will be contacted. The data extraction process will involve 2 reviewers to minimize the chance of errors and bias [21]. Disagreements will be solved through consensus, and if that is not achieved, a third reviewer will provide the final decision. Reviewers will use the Rayyan [20] software to access the full texts of eligible studies and register the information extracted in a Microsoft Excel spreadsheet file.

## Results

A narrative synthesis will be produced to summarize the extracted data and present a comprehensive overview of the methods for the clinical validation of digital endpoints. The study will be reported following the “Preferred Reporting Items for Systematic Reviews and Meta-Analysis: Extension for Scoping Reviews (PRISMA-ScR)” [19]. The search strategy and selection process results will be described, and a PRISMA-ScR flow diagram will be presented [23]. Moreover, the results relevant to this scoping review’s objectives and research question will be described [23], namely, the methods for the clinical validation of digital endpoints, structured around the patterns identified per device, digital endpoint, and clinical application goal. Findings will be compiled in tables and figures where appropriate. We will also identify gaps in the evidence to inform areas of future research.

## Discussion

This scoping review will map the methods for the clinical validation of digital endpoints. It will be unique in its breadth since it will comprise digital endpoints collected from several devices and not focus on a specific disease area. However, we expect to be able to derive more robust trends and patterns of clinical validation of digital endpoints generated from movement, physiological (electrical, optical, and imaging), and biochemical sensors.

Digital clinical endpoints are a new and rapidly growing research field [24]. Research studies assessing the clinical validity of digital clinical endpoints do not accompany the volume of studies reporting the development of digital endpoints [24], likely due to clinical validation being the last phase of the V3 framework, following verification and analytical validation. As research on digital clinical endpoints is a new field, the initial focus is the development phase. Moreover, it has been reported that deployment studies have been conducted without prior clinical validation, therefore not establishing fit-for-purpose and trustworthiness [24]. This can result in either resource wastage or harm [24]. The lack of a standardized methodology is likely a significant reason for the reduced proportion of clinical validation studies of digital endpoints. Without a well-defined and standardized method, more effort and time are required for each study. Hence, patterns must be identified so researchers can rapidly have a reference to guide clinical validation studies of digital clinical endpoints.

There is an asymmetry in the number of publications for body functions, diseases, or conditions, with a predominance of studies assessing digital endpoints derived from movement, physiological (electrical), physiological (optical and imaging), and biochemical sensors [24]. Those endpoints are most commonly used to answer clinical questions and are most common among studies promoted by pharmaceutical companies to support medical product development [11,24,25]. Thus, we may be able to examine better trends and patterns of digital endpoints. We may also be able to identify gaps in the literature, informing the need for further research in specific types of digital endpoints.

This scoping review study will conform with the recommended standards for conducting scoping reviews, which contributes to a robust methodology as well as transparency and reduced risk of bias. However, some limitations are expected. First, we will not undertake a methodological appraisal or risk of bias assessment of the studies included in the scoping review, as that neither is recommended in scoping reviews [21] nor would be adequate for all sources of evidence we are expecting to retrieve (eg, letters, guidelines, website, or blog posts). However, we are aware that the lack of methodological appraisal in scoping reviews is not consensual [26], with some authors defending that it prevents identifying gaps in the literature regarding the low quality of research and using the results to make recommendations for policy or practice [26]. Second, there is a heterogeneity of terms used to describe endpoints generated by digital technology. We included several terms in our search strategy, informed by a pilot search in PubMed we have conducted, but more may be needed to capture all existing studies exhaustively. In addition to that, digital endpoints can be identified using terms related to the technology (eg, inertial sensors, smartwatch, and smartphone), disease (eg, rheumatic diseases), or bodily function (eg, sleep, gait, and hemodynamic). We attempted to create an extensive list of appropriate search terms for all available technologies and bodily functions. Still, the search strategy retrieved a colossal number of results, making a scoping review unfeasible. Following expert advice, we maintained only some of the terms related to technology and the terms related to digital endpoints, expecting a low possibility that additional information of interest may be missed.

We believe this work will be relevant to various researchers, including those designing and developing clinical studies using digital endpoints and those involved in applications for marketing approval of either drugs assessed in clinical trials using digital endpoints or developing digital health technologies to collect data remotely and seeking regulatory approval. It will also inform health care professionals supporting the modernization and acceleration of clinical trials and those interested in integrating digital endpoints in their clinical practice. The study findings could further be used by regulators developing regulations and guidelines for clinical trials and medical devices. Therefore, we plan to disseminate the results of this scoping review through publication in an international peer-reviewed scientific journal and present the main findings in a workshop of the European project COTIDIANA [27] to a diverse group of stakeholders.

## Appendix

Multimedia Appendix 1 Search strategy.

## Abbreviations

ACM: Association for Computing Machinery
CENTRAL: Cochrane Central Register of Controlled Trials
IEEE: Institute of Electrical and Electronics Engineers
JBI: Joanna Briggs Institute
PRISMA-ScR: Preferred Reporting Items for Systematic Reviews and Meta-Analysis: Extension for Scoping Reviews

## Data Availability

Data sharing does not apply to this paper, as no data sets were generated or analyzed during this study.
