# Supplementary material for: Methods for the Clinical Validation of Digital Endpoints: Protocol for a Scoping Review Abstract
Source: JMIR Res Protoc. 2023 Oct 26;12:e47119. doi: 10.2196/47119 (PMC10636620; doi:10.2196/47119)
Supplement: Multimedia Appendix 1 [file resprot_v12i1e47119_app1.docx]

## Search Strategy

Searches to the databases will adhere to the general following structure: (digital endpoints) AND (validation-area terms). The search strategy for MEDLINE (PubMed) is as follows (Table A1).

**Table A1.** Search strategy for MEDLINE (via PubMed).

| String. No. | Query | Results |
| --- | --- | --- |
| #1 (digital endpoints terms) | "digital endpoint*" [Title/Abstract] OR "digital endpoints" [Title/Abstract:~2] OR "digital endpoint" [Title/Abstract:~2] OR "digital measure*" [Title/Abstract] OR "digital measures" [Title/Abstract:~2] OR "digital measure" [Title/Abstract:~2] OR "novel endpoint*" [Title/Abstract] OR "novel endpoints" [Title/Abstract:~2] OR "novel endpoint" [Title/Abstract:~2] OR "digital biomarker*" [Title/Abstract] OR "digital biomarkers" [Title/Abstract:~2] OR "digital biomarker" [Title/Abstract:~2] OR "digital outcome*" [Title/Abstract] OR "digital outcomes" [Title/Abstract:~2] OR "digital outcome" [Title/Abstract:~2] | 2509 |
| #2 (validation-area terms: clinical prediction)^1^ | validat* [Title/Abstract] OR "clinical prediction" [Title/Abstract] OR predict* [Title/Abstract] OR scor* [Title/Abstract] OR observ* [Title/Abstract] OR predictive value of tests [MeSH Terms] OR observer variation [MeSH Terms] | 7,188,542 |
| #3 (validation-area terms: diagnosis)^2^ | sensitiv* [Title/Abstract] OR sensitivity and specificity [MeSH Terms] OR diagnos* [Title/Abstract] OR diagnosis [MeSH Terms] OR diagnostic* [MeSH Terms] OR diagnosis, differential [MeSH Terms] | 12,029,131 |
| #4 (validation-area terms: prognosis)^3^ | prognosis: incidence [MeSH Terms] OR mortality [MeSH Terms] OR follow up studies [MeSH Terms] OR prognos* [Title/Abstract] OR predict* [Title/Abstract] OR course* [Title/Abstract] | 4,044,100 |
| #5 (Final) | #1 AND (#2 OR #3 OR #4) | 1895 |

^1^ Search Filter for MEDLINE in PubMed Syntax for Clinical Prediction Guidelines maximizing sensitivity (Sen 96%, Esp 79% and Acc 79%), available at [Health Information Research Unit - HIRU ~ Search Strategies for MEDLINE in Ovid Syntax and the PubMed translation (mcmaster.ca)](https://fraunhoferportugal-my.sharepoint.com/personal/silvia_rego_fraunhofer_pt/Documents/Desktop/Protocol_Scoping_Review_COTIDIANA%2010082023%20(RH).docx#Reviews)

^2^ Search Filter for MEDLINE in PubMed Syntax for Diagnosis maximizing sensitivity (Sen 99%, Esp 74%, Acc 74%), available at [Health Information Research Unit - HIRU ~ Search Strategies for MEDLINE in Ovid Syntax and the PubMed translation (mcmaster.ca)](https://fraunhoferportugal-my.sharepoint.com/personal/silvia_rego_fraunhofer_pt/Documents/Desktop/Protocol_Scoping_Review_COTIDIANA%2010082023%20(RH).docx#Reviews)

^3^ Search Filter for MEDLINE in PubMed Syntax for Prognosis maximizing sensitivity (Sen 99%, Esp 80%, Acc 80%), available at [Health Information Research Unit - HIRU ~ Search Strategies for MEDLINE in Ovid Syntax and the PubMed translation (mcmaster.ca)](https://fraunhoferportugal-my.sharepoint.com/personal/silvia_rego_fraunhofer_pt/Documents/Desktop/Protocol_Scoping_Review_COTIDIANA%2010082023%20(RH).docx#Reviews)
